# Workshop on professionalism and professional identity formation for newly recruited faculty at a healthcare university: lessons learnt

**DOI:** 10.3389/fmed.2025.1544761

**Published:** 2025-05-19

**Authors:** Himanshu Pandya, Jagdish Varma, Sarmistha Ghosh, Dinesh Kumar, Anusha Prabhakaran, R. Harihara Prakash

**Affiliations:** ^1^Department of Medicine and Medical Education, Pramukhswami Medical College, Bhaikaka University, Anand, India; ^2^Department of Psychiatry, Pramukhswami Medical College, Bhaikaka University, Anand, India; ^3^Department of Health Professions Education, Bhaikaka University, Anand, India; ^4^Department of Community Medicine, Pramukhswami Medical College, Bhaikaka University, Anand, India; ^5^K M Patel Institute of Physiotherapy, Bhaikaka University, Anand, India

**Keywords:** faculty development, health professions education, professional identity formation, professionalism, workshop

## Abstract

**Background:**

Healthcare is inherently human-centered, and professionalism is crucial for improving healthcare systems. Traditionally developed through role modeling, professionalism now necessitates explicit teaching. Despite the inclusion of professionalism-related competencies by Indian regulatory bodies in medicine and nursing, its structured teaching remains limited. To address this, we introduced concepts of Professionalism and Professional Identity Formation (PIF) to newly joined faculty at our university, following established frameworks. This report details the workshop process and key lessons learned.

**Methods:**

A six-member core faculty team with expertise in health professions education conducted a daylong workshop. The morning session focused on contemporary perspectives on professionalism, while the afternoon session addressed PIF. Anonymous post-session feedback was collected via Google Forms, with quantitative responses rated on a five-point Likert scale and qualitative feedback analyzed using Braun and Clarke’s six-phase thematic analysis.

**Results:**

Three workshops were held in June 2024 for faculty who had joined over the past 2 years. Attendance across the three sessions was 25, 23, and 24 participants, respectively, from medicine, physiotherapy, nursing, and allied health disciplines. Response rates for feedback were 80% (*n* = 20), 43% (*n* = 10), and 45% (*n* = 11). Fifty percent (*n* = 21) reported high satisfaction, 41% (*n* = 16) moderate, and 7% (*n* = 3) low. Participants noted shifts in their perceptions, recognizing professionalism as a learned skill rather than an inherent trait. Key takeaways included the significance of effective communication, empathy, and resilience in shaping professional identity. In terms of educational impact, participants intended to model professionalism, reinforce positive behaviors, and explicitly integrate PIF into their teaching. Proposed strategies included experiential learning, structured orientation, and active learning methods. The workshop was appreciated for its interactive elements, such as group discussions, case-based learning, and open forums. However, areas for improvement included better time management, concise delivery, enhanced multimedia use, and more structured engagement. Participants also suggested extending the workshop to resident doctors and a mixed audience of residents and consultants.

**Conclusion:**

The workshop effectively conveyed professionalism and PIF concepts. Future iterations could benefit from refinements in structure and delivery, as well as audience expansion. Our experience may guide similar faculty development initiatives in other healthcare institutions.

## Background

Since healthcare is essentially a human activity in midst of countless interactions guided by overarching ethos or value sets, professionalism and professional values have an important role in improving healthcare systems (1). Earlier, professionalism was considered to be mainly developed through exposure to role models. However, experts in health professions education have argued in favor of explicit teaching of professionalism (2). Erstwhile Medical Council of India (MCI) introduced AETCOM module in 2019 as part of Competency Based Medical Education (CBME) for MBBS students (3). Indian Nursing Council (INC) has listed competencies of professionalism, professional values and ethics including bioethics for B.Sc. Nursing students in its revised regulations and curriculum published in 2021 (4). While healthcare practitioners are required to adhere to codes of practice mandated by regulatory bodies, little attention is paid to the domain of professionalism during post-graduate training and Continuous Professional Development (CPD) activities organized by institutions providing health professions education and professional bodies, respectively. Healthcare practitioners therefore may not know what exactly professionalism means. Lesser et al. have proposed behavioral and systems view of professionalism as a practical approach to improve the delivery of healthcare (4). In addition to discourse on healthcare professionalism, the identity discourse is also coming to the fore, that emphasizes the importance of not just doing but also being. With the publication of Carnegie foundation report in 2010 on the future of medical education, the emphasis has moved from teaching of professionalism to supporting identity formation (5). In view of this shift, faculty development to promote Professional Identity Formation (PIF) becomes crucial strategy to facilitate the process of becoming and being a healthcare professional. The core purpose of our university has focus on providing modern and professional healthcare and resources (6). Therefore, recognizing the gap in structured training on Professionalism and PIF in postgraduate education and faculty development, we conducted this workshop to introduce these concepts to newly joined teaching faculty at our university. This initiative aimed to equip them with the knowledge and strategies needed to model and foster professionalism among learners. We report the process and lessons learned from a series of workshops.

## Methods

### Design

We adapted conceptual frameworks suggested by Levinson et al. and Cruess et al. for designing the workshop (2, 7) (Supplementary material 1, 2). Workshop activities were designed based on theories of cognitivism, constructivism, and social learning (8).

### Setting

We conducted three iterations of the workshop on “Professionalism and PIF” in June 2024 at Bhaikaka University, a healthcare-focused university campus located in the semi-urban region of the state of Gujarat in western India. The university hosts a diverse group of undergraduate and postgraduate students across disciplines such as medicine, nursing, physiotherapy, public health, medical technology and allied health sciences.

### Recruitment process

The inclusion criteria for participants of the workshop was recruitment over the previous 2 years across the constituent institutes of Bhaikaka University. There were no exclusions criteria. This group was purposefully selected as many of them might represent early-career faculty transitioning into academic roles, making them well-positioned to benefit from Faculty Development Program on Professionalism and PIF.

### Planning of the workshop

A day-long workshop was developed by a core group of six faculty with expertise in health professions education and experience in conducting workshops on professionalism and PIF (9). Expected learning outcomes of the workshop were to: (1) describe behavioral and system approach to professionalism; (2) articulate the advantages of viewing professionalism as a multifaceted competency; (3) describe attributes and activities associated with personal wellbeing and resilience; (4) understand the concepts of personal and professional identity and socialization; (5) identify factors influencing socialization; and, (6) discuss personal and institutional strategies to support PIF. Faculty guides were prepared for the sessions. PowerPoint presentations were prepared to guide the flow of the sessions, along with instructions for small group activities. Narratives were provided as handouts to respective groups (7). While no prereading material was shared with the participants, post workshop handout on important concepts with suggested readings, was shared through WhatsApp group created for the participants.

### Implementation of the workshop

Sitting arrangement for participants of the workshop included four tables arranged in a C-shape. Morning session of the workshop focused on newer perspectives about professionalism and the afternoon on PIF. Boxes 1, 2 show the structure of the workshop including the scenarios developed as physical handouts. The workshop featured a series of small group discussions, each followed by sharing of insight by each of the four groups in the large group. A facilitator guided the discussion for deeper understanding of newer perspectives of professionalism and concepts of PIF. For the session on Professionalism, each narrative (Supplementary material 3) had structured questions for the group to debate and present the resulting insight with the large group facilitated by one of the facilitators. For the session on PIF, group activities (Box 2) were followed by presentation of groups’ insight with the large group facilitated by one of the facilitators. In the final wrap-up, participants were encouraged to ask questions and clarify concepts of professionalism and PIF.

BOX 1 **Activities of the morning session on professionalism.**

*Introduction*: Icebreaking, workshop objectives.

*Small group work*: participants’ discussion on four different scenarios* to develop viewpoints.

*Large group discussion*: participants’ viewpoints shared in large group in serial manner for scenario one to four, each discussion followed by introduction of newer assumptions about professionalism by facilitator.

*Scenarios were adapted from Levinson et al. and focused on professionalism challenges and lapses in the clinical environment followed by questions (7).

BOX 2 **Activities of the afternoon session on professional identity formation.**

*Group activity 1*: Facilitator shared his personal and professional identities to facilitate understanding of the group work. Participants reflected on their own personal and professional identities and shared with a colleague detail about one of their identities. Subsequently, they shared their insights about identities in the large group. Facilitator defined identity, professional identity formation, physician’s professional identity, socialization and communities of practice.

*Group activity 2*: Participants reflected on the factors that have influenced their own professional identity and which might influence the identities of their learners. Facilitator elaborated the concept of socialization and factors that influence professional identity formation.

*Group activity 3*: Participants identified personal and institutional actions to support professional identity formation in their institution. Facilitator elaborated on the principles of developing strategies to support professional identity formation.

*Wrap-up*: through questions and clarifications.

Post-session feedback was collected using an anonymous google form. Feedback form included questions on degree of satisfaction with the workshop (Table 1). Three open-ended questions were included to elicit participants’ opinions regarding the changes they would like to bring in their practice with students, to find out their key take-home messages and what did they find most interesting. The participants were also requested to provide any additional comments regarding the overall conduct of the workshop. Demographics were collected from attendance sheet for the workshop.

**Table 1:** Questions asked on the feedback form.

No.	Quantitative items	Rating scale
1	What is your overall satisfaction in the workshop?	3-point rating—High, Moderate, Low
2.	How satisfied were you with the logistics? Mode of Delivery Introduction to the session- Ground rules Wrap up Flow of event within session Duration of Workshop Session Content a) Pre reading materials b) Materials shared during session c) Hands on exercises—small group d) Follow up discussions in large group	5-point Likert scale 1 = least satisfied 5 = most satisfied
	Qualitative questions	
1	What changes are you likely to bring in your practice with students after this workshop?	Open ended
2	What were your key take away messages from this workshop?	Open ended
3	What did you find most interesting?	Open ended
4	What are your other comments about the overall conduct of the workshop?	Open ended

### Analysis

The quantitative questions were analyzed using descriptive statistics (frequencies and percentages). The responses to the qualitative questions were analyzed thematically using Braun and Clarkes’ six-phase approach (10). Phrases were used as the units of analysis. The descriptions of the themes are provided in the results section.

## Results

**Figure 1:**
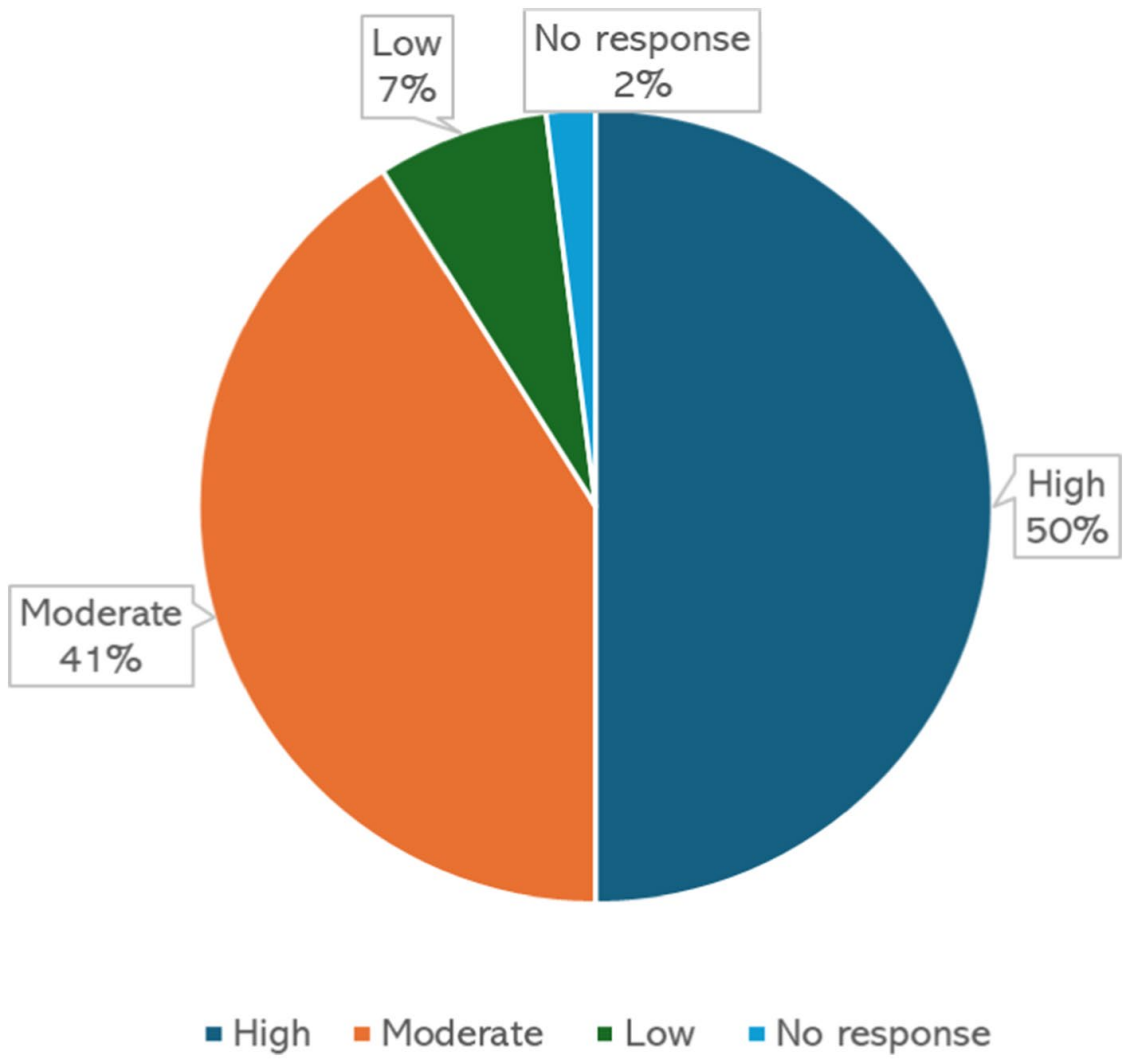
Level of satisfaction of all the participants from all 3 iterations.

The first, second and third workshops were attended by 25, 23, and 24 participants, respectively. Overall, 38 females and 34 males representing junior (*n* = 71) and middle (*n* = 1) level faculty participated in the three workshops. The participants were from medicine, physiotherapy, nursing, and allied healthcare disciplines. The response rates were 80% (*n* = 20), 43% (*n* = 10), and 45% (*n* = 11) in first, second and third workshops, respectively. Figure 1 shows the levels of satisfaction of the participants. The following themes emerged from the qualitative analysis.

### Theme 1: changes in perceptions about personal and professional identity

Participants reflected on the impact of the workshop on their understanding of personal and professional identity. They recognized a shift in their perception of professionalism, personal identity and professional identity. The participants identified following key areas of understanding: assumptions about professionalism and role of effective communication and empathy. The following subthemes, supported by representative quotes, illustrate their insights.

#### Subtheme 1.1—new assumptions about professionalism

Participants developed new perspectives on professionalism, recognizing it as a learned skill rather than an inherent trait. They emphasized the importance of structured education over punitive measures in shaping professional identity. Additionally, they highlighted the role of socialization in forming professional values and distinguishing between personal and professional identity. The following quotes illustrate these evolving assumptions about professionalism:

*“Professionalism needs to be taught and not caught.”*

*“Although it’s caught but professionalism should be taught. Education is better over punishment…Socialization is where identities are formed.”*

*“Professionalism is a skill acquired over time and with experience.”*

*“Educational approach needs to be taken first rather than punitive approach.”*

*“Learnt about the difference between professional identity and personal identity.”*

#### Subtheme 1.2—the role of communication and empathy in professional identity

The participants acknowledged the essential role of communication in fostering inclusivity, compassion, and mutual understanding. They recognized that effective communication not only strengthens professional relationships but also contributes to overall professional growth. The following quotes highlight their commitment to enhancing their communication skills and creating a supportive work environment:

*“I will strive to improve my communication skills by actively listening, articulating my thoughts clearly, and using appropriate body language.”*

*“Creating a supportive, inclusive environment is crucial for professional growth.”*

*“I learned how to navigate challenging situations with integrity and foster positive relationships with colleagues and clients.”*

### Theme 2: anticipated changes in educational practice

In response to the question “What changes are you likely to bring in your practice with students after this workshop?,” thematic analysis revealed two themes about changes in their teaching practices and interactions with students.

#### Subtheme 2.1—professional development and identity formation

Participants recognized the need for a more explicit approach in fostering students’ professional identity. They emphasized modeling professionalism, reinforcing positive behaviors, and guiding students in developing self-awareness regarding their professional growth. By actively demonstrating professionalism and appreciating good practices, they aim to strengthen students’ professional socialization.

*“I will use explicit approach while doing good practices, Appreciate good behaviour and practices of students in front of all students.”*

*“Teaching them the importance of professionalism.”*

*“Train students to identify their strengths and how they can turn their behaviours into professional identity.”*

*“The behaviour attribute, corrective and preventive action, telling them first the reason, and imbibing the professional identity.”*

#### Subtheme 2.2—teaching strategies and methodologies

Participants highlighted their intent to modify teaching methodologies to better instil professionalism. They expressed a commitment to using a variety of pedagogical approaches, emphasizing experiential learning, observations, and active learning strategies such as seminars and workshops. Additionally, they acknowledged the need for structured orientation sessions to introduce students to professional expectations early on.

*“I will try to make them learn through observations, seminars, or workshop and will arrange session on orientation regarding professionalism.”*

*“Foster a classroom culture that values respect, empathy, and inclusiveness.”*

*“Provide regular, detailed, and constructive feedback to students on their performance and professional behaviour.”*

*“Maintaining discipline and finding the cause whenever there is a lapse in professionalism.”*

#### Theme 3—overall perception of the workshop

Many participants expressed positive feedback, indicating that the workshop was “good” or “interactive.” The general sentiment was that the content was relevant and useful, as several respondents appreciated the structure and coverage of topics. Many participants found the discussions and deliberations particularly valuable and appreciated the interactive sessions namely group discussions, case-based learning, and open forum.

*“Overall good.”*

*“Session was good.”*

*“This training should be done for all resident doctors, including first-year.”*

*“Session was very interactive.”*

*“All the facilitators facilitated the session in a very good manner and their discussion was to the point.”*

Several participants provided neutral feedback, including responses such as “No,” “None,” or “No comments.”

### Theme 4: suggestions for improvement

Participants recommended ways and means to enhance the workshop experience. While they appreciated most aspects of the workshop, multiple areas of improvement were highlighted, namely related to delivery, content format, and engagement strategies. Each of the subthemes also shows the representative quotes to justify their statements,

#### Subtheme 4.1—time management issues

Some of the participants found the session lengthy. The participants suggested that structured time slots and short breaks in between talks could be introduced to avoid fatigue and ensure maximum engagement.

*“Sessions should not go on for more than an hour. Everyone may not like.”*

*“…I found there were issues with time management.”*

*“Some of us got lost at times.”*

*“include some breaks with interesting activities.”*

#### Subtheme 4.2—delivery style

The participants appreciated the overall delivery style, however few suggested to make sessions smoother and more engaging. They recommended concise delivery using different instructional methods to satisfy varied learners.

*“Make it smooth and sharp, otherwise it’s completely perfect.”*

*“The way of delivering the session in interactive manner has been great influence for me.”*

*“Session was very interesting and beneficial, content also good.”*

*“I personally feel that, it was good session with debates and discussions.”*

*“Reduce slide content.”*

#### Subtheme 4.3—use of visual aids and interactive elements

The participants emphasized the need for more multimedia content to make sessions more engaging. Incorporating interactive elements such as videos, infographics, and case studies to make learning more dynamic. Few participants felt that humor and interactive activities should have been introduced to maintain interest and involvement and create a more relaxed learning environment.

*“Lecture should include more images or videos.”*

*“Subject glossary or words used in this kind of workshop may be taught with examples, images, etc.”*

*“It should include more of photos rather than only written information.”*

*“Humor can free up boredom in these kinds of sessions.”*

#### Subtheme 4.4—audience for future workshops

Participants suggested that this workshop should be planned and offered to a broader audience, with specific reference to the resident doctors. They also opined that a mixed group of residents and consultants should be offered such workshops together.

*“This workshop should also be delivered to resident doctors.”*

*“More of such sessions should be taken, involving residents and consultants together.”*

## Discussion

The participants’ feedback suggests that the workshop was effective at Kirkpatrick Model Levels I and II, achieving high participant satisfaction (reaction) and demonstrating evidence of learning (knowledge acquisition). The participants’ insights and takeaways aligned closely with the workshop’s intentions, particularly regarding professionalism and PIF, indicating that the workshop effectively fulfilled its objectives.

The conceptual framework suggested by Levinson et al. provided a practical approach to healthcare professionalism by framing professionalism as a set of observable and demonstrable behaviors (7). The approach helped in shaping participants’ views about professionalism. An important learning among the participants was regarding the need for explicit teaching of professionalism and educational approach to handling professional lapses. They realized that professionalism is a set of knowledge, skills and behaviors acquired over time through experiences of navigating challenging situations. Through guided activities, participants gained insight into how professional identity is shaped through socialization. They recognized that professionalism is learned rather than a matter of innate character. With the change in perspectives about professionalism and insight about PIF, the participants expressed motivation to refine their classroom culture and pedagogical approaches.

Key factors facilitating participants’ learning included interactive group discussions, reflective learning activities, and a balance of theory with practical examples. However, time management emerged as a challenge. Some participants felt that sessions were too long, possibly due to cognitive fatigue from dense content. They recommended structured time slots, short breaks, and the inclusion of humor or interactive elements to maintain engagement. While participants appreciated the overall delivery style, several of them recommended refining instructional methods to cater to varied learners. Suggestions included reducing slide content, integrating multimedia such as videos and images.

The participants also expressed interest in expanding the workshop’s reach to resident doctors and consultants. Tailoring sessions for different faculty roles—including early-career educators, senior faculty, and clinicians—could improve relevance and engagement.

The findings align with prior research emphasizing the need for explicit professionalism education (11). The participants’ responses indicate a shift in perceptions and a willingness to integrate new insights into their teaching practices.

This study highlights the effectiveness of a structured faculty workshop in fostering professionalism and supporting PIF. They were motivated to change their educational practices in the light of new insights. The study also indicated that institutions need to integrate professionalism training into ongoing faculty development programs with case-based interactive sessions to enhance engagement and retention.

Based on the feedback responses, a number of lessons have been learned which can be considered for future workshops on similar topic (Box 3). By implementing these changes, future workshops can become more effective, ensuring higher engagement and improved learning outcomes. Continuous evaluation and adaptation based on participant feedback will act as a key to optimizing future training sessions.

BOX 3 **Summary of recommendations for future workshops.**

1. Sessions need to be more well-structured, concise, modular, engaging, and explanatory, with defined time limits and frequent breaks.

2. Sessions to include more visual content with use of use of images, videos, and real-life examples to support key concepts and maintain participant interest and create a more dynamic learning experience.

3. The workshop is to be planned in a suitable format to be offered to residents and consultants of various health professions, to broaden its impact, promoting knowledge-sharing across different levels of expertise.

While many participants actively engaged in providing feedback, response rates in the second and third workshops were lower (43 and 45%). This indicates that some participants might be non-committal about their reactions to the workshop. It may also suggest that a few participants found the workshop unremarkable or not useful to their needs. The variability in response rates across workshop iterations poses a limitation, as it may affect the interpretation of participant experiences.

Additionally, since the study was conducted within a single institution, the generalizability of findings are limited. Future studies should consider multi-institutional evaluations and implement strategies to ensure more consistent response rates.

## Conclusion

This workshop successfully enhanced faculty understanding of professionalism and PIF. Educators can adapt the framework used by us for teaching-learning of professionalism and PIF for undergraduate and post graduate students of health professions. Future research should examine the long-term impact of the faculty development workshop on professionalism and PIF on teaching behaviors and clinical practice. Providers of Continuous Professional Development (CPD) activities can also use this framework for practitioners.

## Data Availability

The raw data supporting the conclusions of this article will be made available by the authors, without undue reservation.

## References

[1] Lesser CS Lucey CR Egener B Braddock CH Linas SL Levinson W. A behavioral and systems view of professionalism. JAMA. (2010) 304:2732–7. doi: 10.1001/jama.2010.1864, PMID: 21177508

[2] Cruess RL Cruess SR Steinert Y. Teaching medical professionalism: supporting the development of a professional identity. New York, NY: Cambridge University Press (2016).

[3] National Medical Commission. Gmraduate medical education regulations. New Delhi: NMC (2019). Available online at: https://www.nmc.org.in/ActivitiWebClient/open/getDocument?path=/Documents/Public/Portal/Gazette/GME-06.11.2019.pdf (Accessed December 4, 2024).

[4] Indian Nursing Council (2021) Revised regulations and curriculum for B.Sc. (nursing) program, regulations, 2020. Indian Nursing Council. Available online at: www.indiannursingcouncil.org/uploads/pdf/1625655521119217089560e588e166460.pdf (Accessed December 2, 2022).

[5] Irby DM Cooke M O’Brien BC. Calls for reform of medical education by the Carnegie Foundation for the Advancement of Teaching: 1910 and 2010. Acad Med. (2010) 85:220–7. doi: 10.1097/ACM.0b013e3181c88449, PMID: 20107346

[6] Bhaikaka University. About university. (2023). Available online at: https://www.bhaikakauniv.edu.in/ (Accessed December 5, 2023).

[7] Levinson W Hafferty FW Lucey CR Ginsburg S. Understanding medical professionalism. New York: McGraw-Hill Education (2014).

[8] Torre DM Daley BJ Sebastian JL Elnicki DM. Overview of current learning theories for medical educators. Am J Med. (2006) 119:903–7. doi: 10.1016/j.amjmed.2006.06.037 17000227

[9] Pandya H Dongre A Ghosh S Prabhakaran A Prakash RH Panchal S. Sensitizing nursing faculty about formation of professional identity: exploration of lessons learnt at a nursing institute in India. Natl Med J India. (2024) 37:145–8. doi: 10.25259/NMJI_705_2023, PMID: 39400001

[10] Braun V Clarke V. Using thematic analysis in psychology. Qual Res Psychol. (2006) 3:77–101. doi: 10.1191/1478088706qp063oa

[11] Zenni EA Turner TL. Planning and presenting workshops that work: a faculty development workshop. MedEdPORTAL. (2021) 11:11158. doi: 10.15766/mep_2374-8265.11158 PMC8110637 34041360

