# Supplementary material for: Workshop on professionalism and professional identity formation for newly recruited faculty at a healthcare university: lessons learnt
Source: Front Med (Lausanne). 2025 May 19;12:1544761. doi: 10.3389/fmed.2025.1544761 (PMC12127412; doi:10.3389/fmed.2025.1544761)
Supplement: Supplementary file 1 [file Table_1.docx]

**Supplement 1 – Framework of Professionalism by Levinson et al**

Based on the framework proposed by Levinson et al., professionalism in medical education

and practice should be approached with contemporary perspectives that reflect its evolving

complexity:

- Emergence of Newer Assumptions: Professionalism is increasingly recognized as context-dependent and responsive to dynamic healthcare environments. This includes a shift from static definitions to those accommodating newer assumptions about professional identity formation and practice (Lucey et al., 2014).
- Systems and Behavioral Approach: Rather than being viewed in isolation, professionalism should be understood through the lens of systems thinking and behavioral sciences. This includes the influence of organizational culture, team dynamics, and structural determinants on professional behavior (Lesser et al., 2010).
- Complex Competency, Not a Character Trait: Professionalism is best conceptualized as a complex, teachable competency composed of multiple domains rather than a fixed character trait (Lucey et al., 2014).
- Observable Behaviors Anchored in Core Values: A practical approach to professionalism emphasizes a set of observable behaviors aligned with four core values—accountability, altruism, integrity, and respect—enabling systematic teaching, assessment, and feedback (Levinson et al., 2010).

**References:**

1. Levinson R, Dewar S, Shepherd S. *Understanding Doctors: Harnessing Professionalism*. London: The King’s Fund; 2010.
2. Lucey CR, Souba WW. *The problem with the problem of professionalism*. Acad Med. 2014;89(8):1018-1024.
3. Lesser CS, Lucey CR, Egener B, Braddock CH 3rd, Linas SL, Levinson W. *A behavioral and systems view of professionalism*. JAMA. 2010;304(24):2732-2737.

**Supplement 2 – Framework of Professional Identity Formation by Cruess et al**

Cruess et al. propose a developmental framework for **Professional Identity Formation (PIF)**

that integrates cognitive, social, and emotional dimensions of learning. It reflects how

individuals *"think, act, and feel like a physician"* over time through structured and experiential

learning:

- Socialization into the Profession: PIF is a social process, where learners internalize the norms, values, and roles of the medical profession through formal, informal, and hidden curricula.
- Role Modeling and Reflection: Observing and interacting with role models in clinical environments plays a critical role. Reflection on these experiences helps learners integrate professional values into their self-concept.
- Stages of Formation: Identity formation is progressive—beginning with the adoption of the identity of a student, then internalizing the values and behaviors of the profession, and ultimately developing a personal sense of what it means to be a professional.
- Supportive Environment: Institutional culture, mentoring, and opportunities for guided reflection and feedback are essential in fostering identity development.
- Intentional Educational Strategies: Educators should create deliberate opportunities, including narrative reflection, professionalism discussions, and feedback, to support identity development.

**References:**

1. Cruess RL, Cruess SR, Boudreau JD, Snell L, Steinert Y. *A schematic representation of the professional identity formation and socialization of medical students and residents: a guide for medical educators*. Acad Med. 2015;90(6):718–725.
2. Cruess RL, Cruess SR, Steinert Y. *Teaching medical professionalism: supporting the development of a professional identity*. Cambridge University Press; 2016.

**Supplement 3 – Scenarios used for session on Professionalism**

**Scenario 1**

During a teaching session on the topic of professionalism, anaesthesia residents narrated a difficult situation which they did not know how to deal with. The residents reported that during operations, one senior surgeon routinely scolded surgical residents, labelling them as incompetent and humiliating them in front of the operating room staff.

Though anaesthesia residents were not themselves attacked by this surgeon, they were upset on behalf of their surgical colleagues and angry at the surgeon for these behaviours. They felt uncomfortable speaking up about the issue, but they wanted these behaviours on the part of surgeon to stop.

1. What professionalism values are violated in this case?
2. How should the anaesthesia residents manage this difficult situation?
3. What skills would the anaesthesia residents need to manage the situation?

**Scenario 2**

Dr. Joshi is a physician practicing in a multispecialty hospital. He has been in practice for twenty years and well respected among his patients, but recently thinking of quitting. He feels that the practice has changed with intense pressure to see more patients in shorter visits, financial incentives for “productivity”, increasing demands for documentation and requirements to measure practice performance in multiple ways.

The hospital has recently decided to adopt electronic medical records completely and go paper free. He is frustrated because of the time he spends getting medical information needed to take care of patients.

1. What factors are influencing the work of the physician in this case?
2. Who should be responsible for addressing the factors influencing the work of this physician and possibly that of other health care providers?
3. How should the factors influencing the work of physicians and others be addressed?

**Scenario 3**

Dr. Patil, a senior clinical faculty, was frustrated by his latest teaching assignment. For years, he has taught clinical skills to medical students in small groups, but this year the curriculum committee added the domain of professionalism, which all clinical teachers were required to teach. It included definitions of professionalism along with expected behaviours for students. Dr. Patil felt that all these did not belong to module of clinical skills, because in his view, “You need not teach this stuff - students will either be professional or they won't. Besides, they should already know how to behave appropriately, by watching their consultants and residents.”

1. What are the views of Dr. Patil about teaching learning of professionalism?
2. What are the views of the group about teaching learning of professionalism?

**Scenario 4**

Dr. Trivedi, the head of Medicine, sighed. He had just received a complaint from one of the ICU nurses who reported that Dr. Shah, one of the medicine residents, screamed at her in the middle of the night when she notified him that an error had occurred and a critically ill 24-year-old patient with bacterial meningitis had missed a dose of antibiotics. Shaking his head, Dr. Trivedi wondered what to do. “If they don't know how to be nice to people by the time they are 26, how am I supposed to teach them? Who let this type of a person into medical college anyway?”

Dr. Shah has felt terrible since the time he shouted at the ICU nurse. He knows he shouldn't have taken his frustrations out on her but he felt like he couldn't help it. Now Dr. Trivedi has called and said he wanted to talk with him about his interaction with the nurse. Dr. Shah worries that his poor handling of this situation will damage his reputation for good.

1. What is Dr Trivedi’s view of professionalism lapse committed by Dr Shah?
2. What approach does the group recommend to manage professionalism lapse on the part of Dr Shah?
3. Justify the approach recommended by the group.
